# A Review on Research Progress of Corrosion Resistance of Alkali-Activated Slag Cement Concrete

**DOI:** 10.3390/ma17205065

**Published:** 2024-10-17

**Authors:** Qiushuang Liang, Xinlu Huang, Lanfang Zhang, Haiyan Yang

**Affiliations:** 1 Chongqing Telecommunications Polytechnic College, Chongqing 402247, China; liangqiushuang69@163.com (Q.L.);; 2 School of Materials Science and Engineering, Chongqing Jiaotong University, Chongqing 400074, China

**Keywords:** alkali-activated slag cement, acid, sulfate, seawater, chemical corrosion mechanism

## Abstract

China is the largest producer and user of Ordinary Silicate Cement (OPC), and rapid infrastructure development requires more sustainable building materials for concrete structures. Portland cement emits large amounts of CO_2_ in production. Given proposals for “carbon peaking and carbon neutralization”, it is extremely important to study alternative low-carbon cementitious materials to reduce emissions. Alkali-activated slag (AAS) cement, a new green cementitious material, has high application potential. The chemical corrosion resistance of AAS concrete is important for ensuring durability and prolonging service life. This paper reviews the hydration mechanism of AAS concrete and discusses the composition of hydration products on this basis, examines the corrosion mechanism of AAS concrete in acid, sulfate, and seawater environments, and reviews the impact of its performance due to the corrosion of AAS concrete in different solutions. Further in-depth understanding of its impact on the performance of concrete can provide an important theoretical basis for its use in different environments and provides an important theoretical basis for the application of AAS concrete, so that we can have a certain understanding of the durability of AAS concrete.

## 1. Introduction

Cement production consumes about 5% of the world’s industrial energy, while each tonne of silicate cement (OPC) requires about 1.5 tonnes of raw materials. The production of 1 tonne of cement emits approximately 0.94 tonnes of carbon dioxide into the atmosphere, mainly from the decarbonisation of calcite in the cement clinker, the combustion process, and the electricity required [1]. Sun et al. investigated the incorporation of blast furnace slag into cementitious materials and found that the mineral composition of blast furnace slag, especially Al_2_O_3_ and SiO_2_, resulted in stronger interfacial bonding with the cement [2,3,4], which could resist the erosion of the material to a certain extent when the interfacial bonding ability of the cement was better. Alkali-activated slag (AAS) cement is a water-hard cementitious material produced by using a strong alkali as the activator and granulated blast furnace slag as the activating material. Concrete is commonly found in a variety of conditions, such as industrial areas, sewers, and marine environments. AAS cements have excellent durability properties in the produced concrete due to the absence of calcium hydroxide, which generally does not produce the expansion-type hydration product calcite [5]. Recently, there has also been research into the use of copper slag in multiphase nano-modified electrically conductive cementitious composites, exploring their electrical conductivity and mechanical properties [6,7]. At present, there are more studies on the basic properties of AAS cement concrete at home and abroad, and there are some studies on its durability aspects, which mainly focus on chemical corrosion resistance, permeability resistance, carbonation resistance, etc. [8,9,10]. This paper reviews the chemical corrosion resistance of AAS cement concrete. Since the corrosion of AAS cement concrete is closely related to its hydration products, the focus is on summarizing the hydration mechanism, acid and sulfate corrosion mechanism and its performance effects, and the effect of seawater on the performance of AAS cement concrete. This review provides key information for further research on the development and application of AAS cement concrete under different conditions.

## 2. Hydration Mechanism of Alkali-Activated Slag Cement

Granulated blastfurnace slag is a calcium-rich reactive material, and its main chemical composition includes 35–50% CaO, 30–35% SiO_2_, 8–15% Al_2_O_3_, and it contains a large amount of FeO, MgO, and TiO [11,12]. Slag has potential water hardness but is difficult to hydrate at room temperature and requires an activator to stimulate its activity and produce gelling properties. Currently, the most commonly used activators are water glass, NaOH, Na_2_SiO_3_, Na_2_SO_4_, Na_2_CO_30_, and their composites. Slag has lower Ca/Si and higher Al/Si than OPC, so there are differences in AAS hydration products. In addition to their unique properties, slag-based materials are also being studied in the context of sustainability, as evidenced in research on the fracture behavior of recycled concrete with waste crumb rubber subjected to elevated temperatures [13,14].

**Figure 1:**
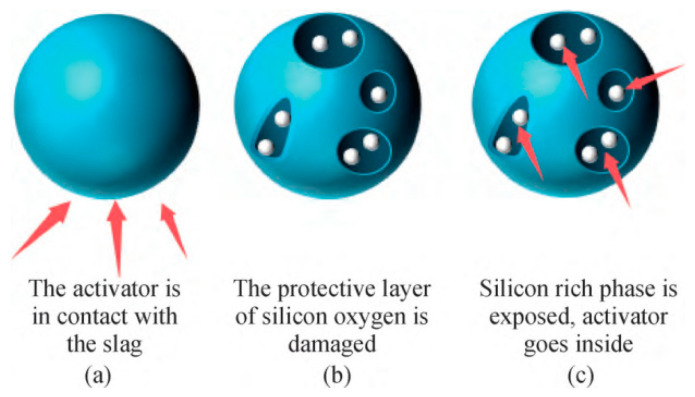
Schematic diagram of failure process of slag phase separation structure [27].

**Figure 2:**
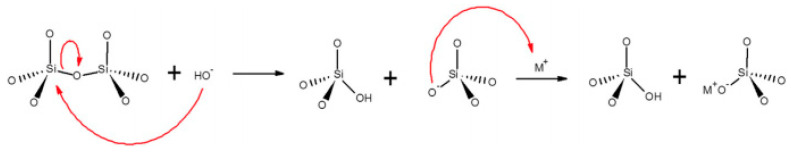
The mechanism of the Si-O-Si bond breakage by the action of OH^−^ [28].

**Figure 3:**
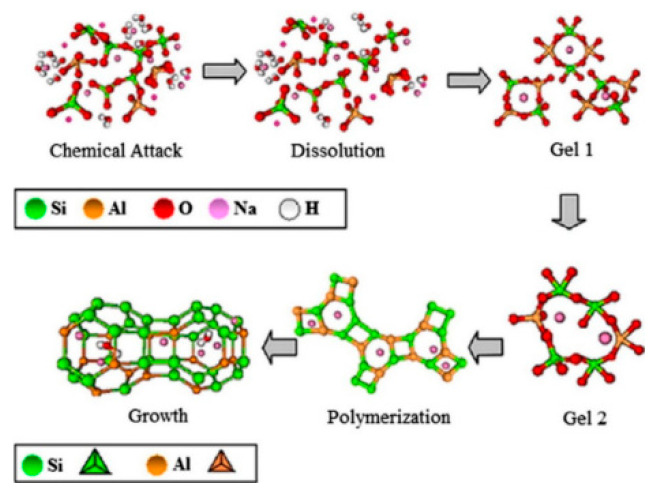
Graphic model proposed for the N-A-S-H gel formation [15].

The hydration process of AAS (Alkali-Activated Slag) is a complex physicochemical process that encompasses multiple stages, including the dissolution of slag particles, ion exchange, precipitation, and crystallization. Under the action of the activator, the protective layer of silicon oxygen is damaged. The Mg-O bond and Ca-O bond in the slag break first due to their weak bond energy, the calcium-rich phase is decomposed, the silica-rich phase is exposed, the activator enters the interior, the slag starts to decompose, and the process of slag fractional phase structural destruction occurs [15,16], as shown in Figure 1. Si-O-Si in the slag glass then decomposes to form the transition compounds -Si-OH and -Si-O- (as shown in Figure 2), but -Si-O- is negatively charged and will combine with positively charged metal cations [17] to form calcium silicate hydrate (C-S-H) gels [18]. As hydration progresses, the C-S-H gel gradually regularises, effective interparticle bonding is achieved, and the slurry structure becomes denser [19]. -Si-O-Al-O bonds and Si-O-Si bonds share the same reaction process to produce [Al(OH)_4_]^−^, [Al(OH)_5_]^2−^, and [Al(OH)_6_]^3−^ to form calcium aluminate hydrate (C-A-H) [17]. As the hydration reaction progresses, the hydration products gradually increase in number and come into contact with each other, forming flocculent structures. These flocculent structures undergo polycondensation reactions, resulting in the hydration products becoming more compact and stable. Alkali-activation also involves the dissolution of aluminum and silicon substances on the surface of the aluminosilicate, and the polymerization of reactive surface groups and soluble substances to form a gel (i.e., gel 1 with high aluminum content is converted to gel 2 with more silicon content), which then continues to develop to form a sodium aluminum silicate hydrate (N-A-S-H) gel [20], and exhibits similar mechanisms to those observed in ternary cementless composites based on red mud, ultra-fine fly ash, and GGBS [21]. As shown in Figure 3, a relatively low Ca/Si (C/S = 0.9–1.2) of calcium aluminum silicate hydrate (C-A-S-H) gel is then produced [22].

In summary, the major hydration products of AAS cement include C-S-H, C-A-S-H, C-A-S, and N-A-S-H [23,24,25]. The minor products change with the type of slag and type of activator [26] and may include hydrotalcite [Mg_6_Al_2_CO_3_(OH)_16_-4H_2_O], C_4_AH_13_, CASH_8_, C_4_ACH_11_, C_8_AC_2_H_24_, etc. [22]. As the hydration product does not produce Ca(OH)_2_, a direct reaction among some chemical substances to produce expansive substances is avoided, and the formation of calcite in the hydration process is also blocked. The C-S-H structure is relatively regular, which can fill the pores well, and with the formation of C-S-H structure being gradually dense, this reduces potential channels through which erosive substances can enter. The formation of the C-A-S-H structure is also more stable and less likely to be damaged, providing the possibility of better chemical corrosion resistance. Based on the polycondensation reaction, the hydration products undergo further crystallization, forming compounds with definite crystalline structures. The formation of these compounds signifies the completion of the hydration reaction and the ultimate hardening of the alkali-activated slag. The hydration mechanism of activated slag is a complex process involving multiple stages such as dissolution of alkali metal ions, hydration reactions of slag, formation and evolution of hydration products, as well as the underlying hydration mechanisms. This process has a significant impact on the properties and applications of alkali-activated slag.

The excitation effects vary significantly with different activators. When sodium metasilicate is used as an activator compared to water glass, the strength development is faster, which is likely attributed to the specific chemical properties of sodium metasilicate. On the other hand, when sodium hydroxide serves as the activator, it may generate a small amount of ettringite or other hydrated aluminates. However, the quantity and types of these products are influenced by various factors such as activator concentration, slag composition, reaction conditions, and so forth.

## 3. Acid Corrosion Resistance of Alkali-Activated Slag Cement Concrete

### 3.1. Mechanism of Acid Corrosion

Acid corrosion is a phenomenon that causes a decrease in the alkalinity of concrete, leading to the decomposition of cement hydration products [29]. Concrete itself is alkaline. Therefore, if the acidity is slight, pockmarks will appear on the surface of the concrete; if the acidity is strong enough and in a large amount, salts (calcium chloride, sodium chloride, etc.) will be produced inside the concrete; the corrosion is more rapid if the acid is mobile. Acid corrosion exists in the environment, e.g., acid rain, rivers in industrial and chemical areas, and microorganisms. Although the hydration product Ca(OH)_2_ is minimal in AAS cement concrete, the presence of alkali activators makes the pH in the pores high nonetheless [30]. In the early stage of acid erosion, there is more OH^−^ in the pores of AAS cement concrete, and when H^+^ enters the pores by osmosis, there can be enough OH^−^ to react with it and keep the pH high [30], thus no obvious degradation can be seen in the early stage [31]; however, with the prolongation of time, the H^+^ dissolves some of the calcium, which in turn leads to the degradation of the gel [32]. Studies have shown that AAS cements leach more Ca^2+^ in acidic environments, and since the slag itself is insoluble in acidic solutions, Ca^2+^ is mainly introduced by raw materials or produced by decalcification of the gel [33,34,35]. In addition, acid produces direct damage to the Si-O-Al bond and de-alumination occurs, leading to changes in the composition and structure of the silicoaluminate network [32]. If the corrosion medium is sulfuric acid, acid corrosion also produces the swelling substance gypsum [36]. The pH of the acidic solution has a great influence on the degradation of AAS cement concrete [37].

**Figure 4:**
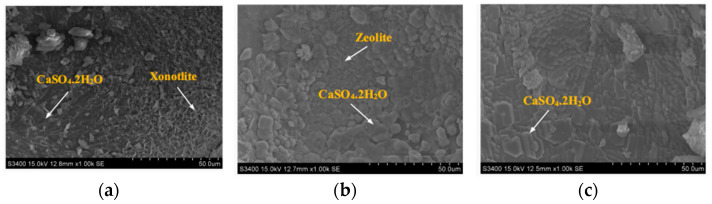
Corrosion morphology of alkali-activated slag cement concrete by biological sulfuric acid [33]. (**a**) CaSO_4_•2H_2_O and Xontlite; (**b**) the surface condition of zeolite; (**c**) the condition of the pore wall.

In addition to chemical sulfuric acid erosion, biological sulfuric acid corrosion also exists. Biological sulfuric acid is caused by microorganisms, and studies have shown that the erosion product is still gypsum [38]. Xie Y [39] showed that the hydration products of AAC before corrosion are hard calcium silica, tobermorite, C-S-H, and zeolite. Figure 4 shows the corrosion morphology of AAC cement concrete; Figure 4a shows hard calcium silica is fused with gypsum; Figure 4b shows that the surface of the zeolite is covered with a layer of gypsum; and Figure 4c shows that the whole pore wall is covered with gypsum, the crystal shape of gypsum is clearly visible, and the product at the original pore wall is covered with gypsum. It can be seen that the calcium ions generated by the decalcification reaction of hard calcium silica are corroded by biosulfuric acid combined with the intruding SO_4_^2−^ to form gypsum; zeolites and other silica-aluminate materials were corroded by biosulfuric acid, which led to the dissolution of aluminum ions. This resulted in the damage of the structure and the loss of the original crystal shapes, and the zeolites were also covered by gypsum eventually. The corroded area swells and cracks, which will exacerbate the leaching of acidic ions and make acidic corrosion more severe.

### 3.2. Effect of Acid Corrosion on Concrete Properties

Acid corrosion begins on the surface of concrete, and the degree of corrosion is related to the pH of the acid solution, the properties and proportion of slag, and the type of acid [38]. Fang Z [40] found that when AAS cement concrete specimens were put into sulfuric acid solution, bubbles were generated on the surface accompanied by rotten egg odor. With the increase in sulfuric acid concentration, the surface of the concrete was chalked severely, and the corrosion was aggravated. At the same time, the longer the exposure time of AAS cement in a sulfuric acid solution, the more serious the strength loss [41]. Lee [42] showed that in alkali-activated slag/fly ash mortar, the larger the slag content, the more detrimental to the resistance to sulfuric acid corrosion, this is because a higher calcium content of slag is more prone to gypsum production, which leads to expansion and associated crack formation [43,44]. In Khan’s study, the samples were immersed in a 3% sulfuric acid solution for 90 days. The results showed that, after 90 days, the compressive strength of all the samples was significantly reduced [45]. AAS concrete showed better corrosion resistance than OPC concrete after 90 days. AAS concrete lost only 21% of its strength after 120 days of exposure to sulfuric acid at pH 1, while OPC-based concrete suffered a 71% loss of strength. OPC exposed to 5% sulfuric acid solution lost 103% of its mass in 100 days, while AASC lost only 10% in 365 days [46].

Ren J [37] studied alkali-activated slag/fly ash mortar in the presence of phosphoric acid [37,44] and found that the degradation depth increased when the slag dosage increased, but the degradation of the alkali-activated mortar slowed down with the extension of erosion time. Additionally, the phosphoric acid solution degraded the mortar more deeply than the mixture of phosphoric acid and sulfuric acid, while the sulfuric acid solution degraded the mortar the least. Similar conclusions were obtained by Jie [47]. The different corrosiveness of the three solutions may be due to the difference in the type and concentration of the acid, or it may be related to the release of H^+^ concentration in an aqueous solution [48]. Zhao W [49] immersed the AAS mortar in acetic acid and sulfuric acid solutions with the same PH, and the surface of the specimens both showed obvious flaking phenomenon after 28 d. The smaller the pH, the rougher the surface of the specimens, and the corrosive effect of acetic acid was the most obvious. It can be seen that acetic acid has a stronger destructive capacity for AAS mortar. If AAS mortar is exposed to hydrochloric acid and nitric acid, hydrochloric acid leads to more obvious quality loss of AAS mortar, which is because hydrochloric acid can react with free Ca^2+^ in AAS mortar to produce the highly soluble salt CaCl_2_ [49]. It has been shown that the mass loss of AAS in a hydrochloric acid solution increases with increasing immersion time [50,51], and changes in mass were more pronounced in samples subjected to acid attack, up to 4.5–5.5%, than in samples immersed in water.

Lloyd [52] compared the corrosive effects of sulfuric acid and nitric acid on AAS cement, geopolymer cement, and calcium aluminate cement by taking them as the objects of the study. It was confirmed that sulfuric acid corrodes AAS cements the slowest and nitric acid corrodes AAS to a lesser depth than sulfuric acid. Ana [53] applied two methods of acid neutralization capacity monitoring and mass loss/consumption of acid monitoring for rapid testing of acid attack resistance of alkali-activated specimens. Teymouri [54] investigated the effect of different mix design parameters on the durability of AAS concrete in a hydrochloric acid solution and the study showed that potassium hydroxide as an alkaline activator in AAS concrete showed higher strength reduction and weight loss in hydrochloric acid solution than sodium hydroxide, and that the lower alkali equivalent gave AAS concrete better acid resistance. At the same concentration of 5%, the compressive strength loss of AAS concrete was highest in H_2_SO_4_, followed by HCl and HNO_3_ [55]. Organic acids such as acetic acid (CH_3_COOH) degrade more than chemical acids at the same pH. Bernal et al. showed that acetic acid at pH 4.5 was more corrosive to AAS than the more acidic nitric, sulfuric, and hydrochloric acids [56]. Nevertheless, Ren et al. demonstrated that AAS is still more stable than OPC in an acidic environment [37].The effect of acid corrosion on concrete properties was studied, as shown in Table 1.

## 4. Sulfate Corrosion Resistance of Alkali-Activated Slag Cement Concrete

### 4.1. Destruction Mechanism of Sulfate Corrosion

Sulfate erosion is one of the important factors affecting the durability of concrete. Heavily salted soil, inland salt lakes, industrial wastewater, groundwater, seawater, and other environments contain a large amount of sulfate, and if concrete exists in these environments, it will be damaged by SO_4_^2−^ erosion [54]. The corrosive destruction of concrete by sulfate is due to a complex combination of physical and chemical actions working together to produce expansive substances (calomel and gypsum) [57]. Since the hydration products of AAS cement concrete are different from those of OPC concrete, the corrosion mechanism differs.

#### 4.1.1. Sodium Sulfate Corrosion Damage Mechanism

**Figure 5:**
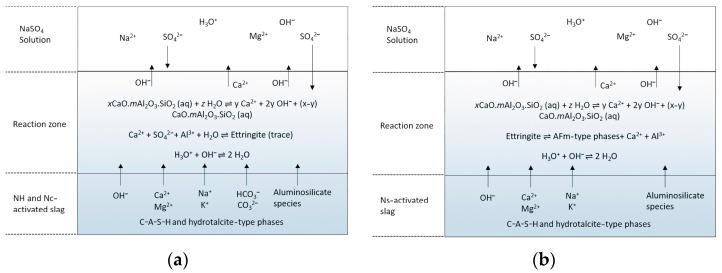
Proposed reaction mechanisms of AAS with Na_2_SO_4_ solution at the exposure surface: (**a**) NaOH and Na_2_CO_3_-activated slag; (**b**) Na_2_SO_4_-activated slag [54].

Ogawa et al. investigated the sulfate corrosion resistance of ground blast furnace slag cement (GGBS) with different contents of calcium sulfate. The sulfate corrosion resistance was evaluated using ASTMC 1012, and multiple mechanisms of sulfate corrosion resistance of GGBS cement were revealed by the analysis of hydration products and sulfate ion intrusion. The results showed that the hydration products in an alkali-stimulated slag cement matrix first reacted with external sulfate ions to generate part of calcium alumina, which reduced the possibility of other internal substances reacting with external sulfate to achieve the effect of sulfate corrosion resistance [58]. Additionally, research on the mechanical and conductive performance of electrically conductive cementitious composite using graphite, steel slag, and GGBS has been conducted, providing insights into the potential applications of GGBS in multifunctional cementitious materials [59]. According to Jin Y [60], the anti-ionic erosion performance of AAS cement concrete is closely related to the composition and structure of the hydration product phase, and the reaction mechanism of AAS cement and sulfate with different activators is different. Under Na_2_SO_4_ erosion, when Na_2_CO_3_ activates (Nc-activated slag) the slag, the presence of carbonate prevents the formation of calcium alumina due to the competition mechanism between carbonate ions and sulfate ions in the formation of calcium alumina; calcium alumina is easily formed when Na_2_SO_4_ activates (Ns-activated slag) the slag, and Na_2_SO_4_ corrosion causes the conversion of calcium alumina to monosulfate. This contradicts the mechanism of phase change of sulfate-containing salts in the OPC system. In the OPC system, calomel is usually formed due to the reaction of the existing AFm phase with the intruding sulfate, whereas for AAS, the AFm is formed by the decomposition of the existing calomel in the surface-exposed portions [61] (as shown in Figure 5). There is also the most common mixture of NaOH and Na_2_SiO_3_ which activates the slag, where the silicate in the activator plays an important role, and the amount of silica in the system increases when used. In addition, it is known that, in the reaction of alkali-activated materials, the alumina of the precursor is more active than silica, and that silica in the silicate activators reacts with the alumina that is initially released from the precursor, thus accelerating the formation of gels [62]. So, when Na_2_SO_4_ erodes, having less Ca^2+^ and Al^3+^ involved in the reaction produces calcite and gypsum swelling products. So, the mixed activated slag of NaOH and Na_2_CO_3_ (NH-andNc-activated slag) is better for Na_2_SO_4_ erosion resistance. Moreover, the structure and composition of C-A-S-H gels depend greatly on the type of activator, and NaOH-activated slag has a higher Ca/Si ratio and a more ordered structure compared to Na_2_SiO_3_-activated slag [63]. The C-A-S-H phase undergoes slight decalcification and de-alumination and promotes the production of trace chalcocite since calcium in the alkali-activated system may undergo dissolution by ion exchange with Na_2_SO_4_ [61,64].

Another study showed that there were Na_2_SO_4_ crystals in the crevices of AAS cement specimens immersed in Na_2_SO_4_ solutions [34]. The study of Rong Z [65] also confirmed that the destruction of AAS cement in Na_2_SO_4_ solutions was due to the infiltration of Na_2_SO_4_ solution into the pores, which formed salt crystals inside to produce volume expansion damage, and the surface of the specimen was gradually peeled off and chalked through the wet and dry cycles. However, with the prolongation of the immersion time, the compressive strength would show a tendency to increase. Analysis of the reasons for this shows that the dissolution and reaction of excess alkali promotes hydration, Na_2_SO_4_ can actually act as an excitatory agent for AAS cements to make the structure denser, which, together with the small amount of calcite and gypsum present [23], produces a filler effect internally to make the microstructure denser [66,67,68]. In addition, chemical corrosion resistance is better due to the lower porosity of AAS than that of OPC and the higher curvature of the pore structure, which provides some inhibition of ion intrusion [69,70].

#### 4.1.2. Magnesium Sulfate Corrosion Damage Mechanism

Compared with Na_2_SO_4_, the erosion mechanism of MgSO_4_ on AAS cement concrete is different. Due to the reaction between Mg^2+^ and C-S-H gel, MgSO_4_ erosion is not simply “sulfate erosion” [71]. In fact, the key factor determining the rate and effect of sulfate attack in alkali-activated systems is the nature of the anions and cations of the attacking medium, with the presence of magnesium ions decalcifying the C-A-S-H, producing gypsum, and leading to degradation of the gel system [64,72]. The mechanism of MgSO_4_ erosion resistance of AAS cement concrete using different activators is different. For NaOH-activated slag cement, in the initial stage, Mg^2+^ reacts with OH^−^ in the pore solution to form hydromagnesite adsorbed on the surface of the hydration product particles, which hinders further erosion. As the OH^−^ in the surface layer is consumed, the buffering effect of the hydromagnesite is gradually lost, and the pH of the surface layer rapidly decreases to 10.5. This is the equilibrium pH of saturated hydromagnesite, and in the absence of the buffering capacity of organic salts, the pH of the surface AAS decreases rapidly, which leads to direct decalcification of the C-A-S-H gel. However, at this pH value, there is no large amount of dealumination, thus the Al-Si ratio in C-A-S-H remains relatively unchanged, and the intruding Mg^2+^ further reacts with the decalcified C-A-S-H to form magnesium silica-aluminate hydrate (M-A-S-H) [61].

**Figure 6:**
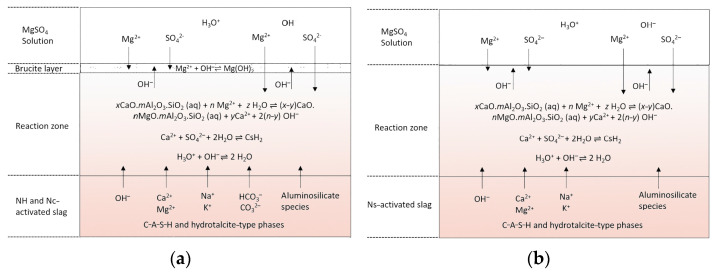
Proposed reaction mechanisms of AAS with MgSO_4_ solution at the exposure surface: (**a**) NaOH and Na_2_CO_3_-activated slag; (**b**) Na_2_SO_4_-activated slag [54].

C-S-H is unstable at a pH below 10 where Mg^2+^ ions are exchanged, and this ion exchange changes the chemical composition and structure of the gel as Mg^2+^ replaces Ca^2+^ in C-S-H to form hydrated magnesium silicate (M-S-H). The Mg^2+^ ions may also be combined with silicate, aluminate, and other constituents of the gel through chemisorption to form new chemical bonds. As the reaction proceeds, precipitates of silicate and magnesium complexes may form in the solution, and these precipitates gradually accumulate inside the concrete to form M-S-H gels. Together, the reaction between the effective Ca^2+^ in the pore solution and the intruding SO_4_^2−^ will produce gypsum (e.g., Figure 6) [61,64]. M-A-S-H, M-S-H, and gypsum are expansive substances, which ultimately cause the destruction of AAS concrete [46]. AAS cements are low in aluminum, and when the hydration reaction produces a C-A-S-H gel, there is no more free Al^3+^ provided to produce calcite. Therefore, there is almost no appearance of calomel in MgSO_4_ corrosion [73,74]. The reaction mechanism of Na_2_CO_3_-activated slag with MgSO_4_ is similar to that of NaOH-activated slag, but the carbonate phase contained in the specimen reacts with MgSO_4_, possibly forming MgCO_3_ and releasing it into the pore solution [61]. For Na_2_SO_4_-activated slag, it is unlikely that a protective layer of hydromagnesite would form due to insufficient available OH^−^, and the subsequent reaction is thought to be similar to that of NaOH-activated slag [61]. In summary, compared to NaOH^−^ and Na_2_CO_3_-activated slag, Na_2_SO_4_-activated slag is less resistant to MgSO_4_ erosion due to the lack of formation of a hydromagnesite protective layer.

### 4.2. Effect of Sulfate on the Properties of AAS Concrete

One of the factors affecting the resistance of AAS cement concrete to sulfate corrosion is the type of sulfate [75], as it depends mainly on the cation. To date, there is a large body of literature comparing the corrosive properties of sodium sulfate and magnesium sulfate erosion on AAS, which are bound to have different degrees of influence on their properties because of the large differences in the corrosion mechanisms of these two sulfates. The effect of sulfate on the properties of AAS concrete was studied, as shown in Table 2. First of all, sodium sulfate erosion affects the surface, reaction products, and strength of AAS cement concrete to varying degrees. The results of Ahmad [76], who immersed alkali-activated slag/fly ash mortar in Na_2_SO_4_, showed that there were no visible cracks or swellings on the surface edges of the mortar specimens, and that the loss of strength in the specimens was only in the range of 1–17%. Li [77] immersed the AAS mortar in a Na_2_SO_4_ solution for wet and dry cycle tests. No gypsum was observed, indicating that AAS mortar has little or no sodium sulfate erosion problems, but the crystallization pressure and diffusion stress of sodium sulfate may cause severe spalling of the surface. The detection and measurement of such cracks are crucial for assessing the durability of AAS cement concrete, and recent research has focused on improving the automation of crack detection, such as the novel visual crack width measurement based on backbone double-scale features proposed in [78], which enhances the accuracy and efficiency of crack detection. When MgO and CaO are used to activate slag, MgO as an activator produces more hydrotalcite and slightly better resistance to sodium sulfate erosion than CaO-activated slag mortars [79]. It has been widely recognized that, in sodium sulfate solutions, the early strength of AAS cement concrete hydration will increase and the late strength will decrease. Jun W [80] also immersed AAS cement in Na_2_SO_4_ solutions to observe its strength development and mass changes, and showed that its compressive strength increased with the increase in erosion time, with the mass remaining basically unchanged. It indicates that sodium sulfate has a contributing effect on its strength development. It has also been shown that the compressive strength still decreases with the continuous extension of erosion time [81,82]. Secondly, the magnesium sulfate erosion process is more complicated. Studies have shown that, after prolonged immersion of AAS cement in a MgSO_4_ solution, although there is no obvious change in appearance, the penetration rate of SO_4_^2−^ ions decreases with the extension in hardening time due to the formation of hydration products filling the internal pores [83], while the compressive strength still decreases with the increase of solution concentration and immersion time [84].

Baščarevć showed [5] that the compressive strength of AAS concrete decreased more significantly in MgSO_4_ solutions than in Na_2_SO_4_ solutions. Hua B [85] showed that in Na_2_SO_4_ solutions there was no change in the surface of AAS specimens, and in MgSO_4_ solutions the reaction between free Mg^2+^ and OH^−^ in AAS produced Mg(OH)_2_ white precipitates attached to the specimen surface. In addition, when Mg^2+^ and SO_4_^2−^ coexisted, it led to shrinkage in the formation of cracks within the concrete, and corrosion increased. Yu H [86] immersed AAS cement into Na_2_SO_4_ and MgSO_4_ solutions and found that the coefficient of expansion increased only slightly (0.176–0.453%), and the microstructure remained intact. Magnesium sulfate erosion caused cracks inside the concrete and reduced its strength, so that the corrosion was more pronounced, while the destructive effect of sodium sulfate erosion decreased.

**Figure 7:**
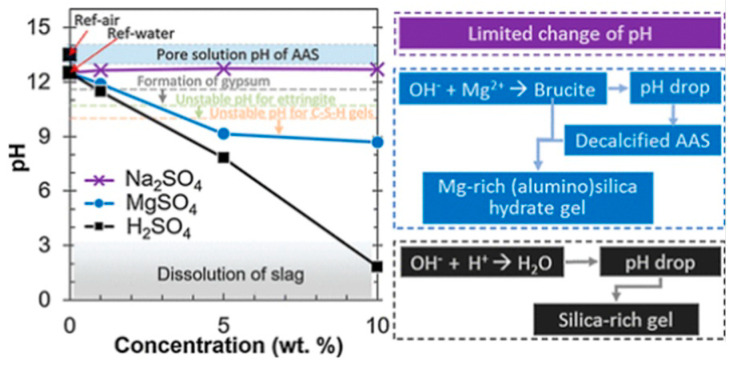
Effect of sulfate on pH of AAS pore solution [79].

In addition to the way the type of sulfate affects the erosion effect, the erosion effect also varies with different sulfate concentrations. Gong [87] found that sodium sulfate with a mass percentage of 1–10% and magnesium sulfate with 1% had less effect on AAS and produced less caliche and gypsum. However, magnesium sulfate with a mass percentage of 5–10% can lead to complete disintegration of the gel, and the magnesium sulfate erosion made the internal production of M-S-H and a large amount of gypsum. These differences are related to the ability of the ions (Na^+^, Mg^2+^, H^+^) to synergize SO_4_^2−^ to change the pH in the pore solution. The effect of Mg^2+^ is greater than that of Na^+^, and it is mainly in the presence of Mg^2+^ that the production of magnesia hydrate lowers the pH so that the decalcification of the AAS produces M-S-H, as shown in Figure 7, so that at the same mass percentage of magnesium sulfate is more able to affect the durability of AAS.

The durability of AAS cement concrete is better than silicate cement concrete due to its denser structure. AAS cement concrete showed better durability than OPC concrete in Na2SO4 solutions [88]. Sheng S [79] observed the products of OPC mortar and AAS mortar eroded by sodium sulfate by XRD and found that the main cause of cracking in OPC mortar was the formation of calcite and gypsum. There were more hydrotalcite-like structures in the erosion products of AAS mortar, whose properties of being able to consume part of the aluminum phase and adsorb sulfate ions hinder the formation of the erosion products such as calcite. Komljenovic [89] studied the changes in strength of slag silicate cement and AAS cement immersed in a Na_2_SO_4_ solution for 90 d. The strength of slag silicate cement increased slightly at 30 d and began to decrease at 60 d. The strength of AAS cement maintained an increase in strength during the test stage. However, the compressive strength of AAS mortar specimens in a 10% MgSO_4_ solution decreased significantly and the loss of strength was greater than that of OPC mortar, which may be attributed to the lack of Ca(OH)_2_ in AAS mortar. This restricts the formation of the protective layer of hydromagnesite, thus leading to a direct attack of Mg^2+^ on the C-S-H structure [46]. Aydn [73] has a different view, suggesting that, in a 10% MgSO_4_ environment, the loss of strength of OPC concrete was greater than that of AAS cement concrete, a large amount of gypsum and calomelite was produced inside OPC concrete and the specimens were damaged, the surface of the AAS concrete did not show any cracking, and crack formation was observed in the region of 20 to 25 μm depth.

## 5. Alkali-Inspired Slag Cement Concrete for Seawater Corrosion Resistance

**Figure 8:**
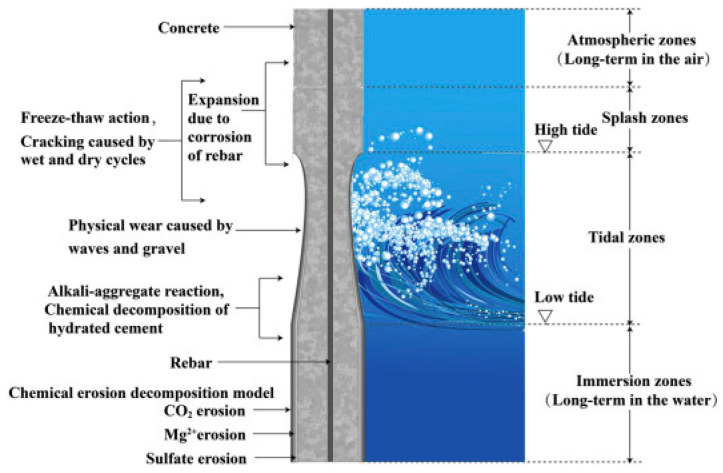
Different degree of corrosion on different parts of concrete structure in marine environment [83].

According to the statistics of 2020, about 40% of the world population lives within 100 km of a coast and 10% live in low elevation coastal zones less than 10 m above sea level [90]. As a result, concrete is often found in seawater environments, such as sea bridges and harbor terminals. The marine environment is a harsh and complex corrosive environment, where components of seawater can be transported into concrete through connecting pores, where the abundance of sulfates and chlorides reduces the durability of the material, leading to the deterioration of the reinforced concrete structure, and affecting its load-bearing capacity [91,92]. Different parts of concrete corrode differently in seawater environments, as shown in Figure 8. The main hydration products of OPC are high-calcium-type calcium silicate hydrate, calcium hydroxide, and calcium aluminate hydrate, and the sulfate in seawater reacts with the hydration products of the cement to produce expansion and cracking. Byung Hwan [93] investigated the effect of relative humidity on the permeability of chloride ions in concrete by determining the chloride ion permeation profiles of concrete specimens, and found that chloride ions were more diffusible in saturated concrete pore solutions, and that the poor moisture connectivity of the pore structure of partially saturated concrete impeded the diffusion of chloride ions. This indicates that the degree of chloride ion diffusion is related to the degree of densification of the material itself. AAS cement concrete hydration products are free of calcium hydroxide and are more dense, which makes them more suitable for application in the marine environment. Shi [94] confirmed that AAS mortar can provide better protection for steel reinforcement. The study by Yin C [95] found that the strength of NaCl-doped AAS cement increased with the increase in dosage, and the degree of slag hydration and C-S-H content increased significantly, which was because the addition of NaCl produced NaOH, which increased the alkalinity of the liquid phase of AAS cement and promoted the further hydration of the slag; however, there was no significant change in the strength of CaCl_2_-doped AAS cement. Therefore, the higher chloride binding capacity of AAS cements provides potential feasibility for the use of seawater as mixing water in marine environments [96]. Studies have shown that replacing fresh water with seawater in concrete can increase the compressive strength of concrete [97]. Mengasini [98] also found that AAS concrete mixed with seawater and cured in a seawater environment had good mechanical properties, and as the time of curing in a seawater environment was extended, the compressive strength increased, reaching 66 MPa at 56 d, which is much higher than that of AAS concrete mixed with fresh water. The compressive strength of AAS concrete cured with fresh water was 7 MPa higher than that of AAS concrete mixed with fresh water and cured with fresh water. This is because the Mg^2+^ contained in seawater reacted with OH^−^ in the alkaline environment to form hydromagnesite, and Ca^2+^ released from the matrix of AAS cement concrete reacted with OH^−^ in the alkaline environment to form Ca(OH)_2_, then reacted with the carbonate in the seawater to form calcium carbonate on the surface of the specimen, so that the generated hydromagnesite and calcium carbonate played a certain protective role for the concrete [99]. However, Li Y [100] showed that cracks appeared in the cross-section of seawater-mixed AAS-cemented concrete when the external temperature increased above 200 °C, and the higher the temperature, the more pronounced the cracks were. Yang S [101] prepared AAS-cemented concrete by substituting seawater and sea sand for freshwater and river sand, and found that there was an effect on the morphology of the hydration products of the AAS-cemented concrete, and that drying shrinkage was slightly increased. Resistance to chloride ion penetration was enhanced, in addition to higher short-term bond strength, interfacial shear stiffness, and shear fracture energy with embedded reinforcement inside. This may be due to the fact that seawater and sea sand accelerate the formation of the C-S-H gel phase [24]. Due to the high chloride content of seawater, much research has been conducted on the degradation of steel in seawater concrete due to corrosion. When seawater is utilized to mix OPC concrete, a significant amount of corrosion occurs in the steel reinforcement [102]. Pitting corrosion of steel in seawater has been found to cause a large number of corrosion pits on the surface of the steel [103]. Jie Liu et al. [104] found that the tensile strength of FRP panels was significantly affected by seawater corrosion, with the strength decreasing in an approximately linear manner with time. Significantly lower capacities were obtained for FRP samples subjected to wet cycle corrosion compared to immersion conditions.

While OPC concrete mixed with seawater presents higher initial strength than with tap water, a significant reduction in strength occurs with age, and after a few years the use of seawater leads to the formation of deeper corrosion pits compared to tap water [105]. Li [106] showed that seawater and sea sand significantly accelerated the setting time of cement with an early strength effect, but harmful components in seawater hindered the later development of concrete strength. This is in line with Mohammed’s view [105]. Chale observed chloride in concrete in the marine environment early on and recommended the use of fly ash with a low water-cement ratio to make concrete more resistant to seawater erosion. The utilization of seawater leads to deeper corrosion pits in concrete compared to ordinary tap water [107]. Sulphates in seawater are mainly sodium sulphate and magnesium sulphate. Sulfates react with cement to form alumina (AFt) and gypsum, and AFt is the main material providing the early strength of cementitious materials [108]. However, AFt and gypsum are extensible, and they gradually grow into the pores at the interface region of the hardened slurry and aggregates. Excessive amounts of AFt and gypsum can easily cause microstructural damage at a later stage, increasing water infiltration channels and reducing the durability of the structure [109]. Typically, tidal environments accelerate concrete corrosion, and Rashad [110] showed that specimens exposed to simulated tidal zones were more severely damaged than specimens fully immersed in seawater. This is in keeping with the damage pattern of OPC concrete. Since encountering tidal environments on the coast is inevitable, even though AAS concrete exhibits good resistance to seawater corrosion, it still struggles to withstand the damage caused by tidal environments. Therefore, studying the corrosion resistance of AAS concrete under tidal conditions is of great significance. AAS concrete corrosion resistance study to seawater, as shown in Table 3.

## 6. Conclusions and Outlook

From the above review and analysis, the following conclusions can be obtained:

(1) Under the action of alkaline activators, the slag is activated and generates hydration products such as C-S-H, C-A-S-H, C-A-H, and N-A-S-H. The hydration reaction process includes the disintegration of slag, the fracture and bonding of functional groups, and the polymerization reaction.
(2) The mechanism of acid corrosion is primarily due to the changes in the gel structure caused by H+, leading to the decalcification of the gel and the subsequent formation of expansive substances. In the case of both chemical and biological sulfuric acid erosion, the destruction of AAS concrete is attributed to the formation of gypsum. Regardless of whether it is chemical action or diffusion, different types of acids have varying degrees of corrosion on AAS cement concrete, with stronger acids causing more severe corrosion. Additionally, a lower alkali equivalent can enhance the acid resistance of AAS concrete.
(3) Sulfate corrosion mainly includes sodium sulfate and magnesium sulfate corrosion. While sodium sulfate corrosion may produce a small amount of gypsum and calcium alumina, magnesium sulfate is more complex compared to sodium sulfate, resulting in hydromagnesite, M-S-H, M-A-S-H, and gypsum, so that the destruction of AAS cement concrete is serious; therefore, AAS cement concrete resistance to sodium sulfate corrosion is better than magnesium sulfate, and compared with the ordinary silicate cement concrete durability, is also more excellent.
(4) AAS cement concrete exhibits good resistance to seawater corrosion. When AAS concrete is immersed in a solution rich in chloride ions, it promotes the development of strength. Furthermore, preparing AAS cement concrete with seawater and sea sand can enhance its mechanical properties to a certain extent. The use of seawater as mixing water has potential feasibility. This conclusion contrasts with that of OPC concrete, making AAS concrete more suitable than OPC concrete for use in coastal environments.

Currently, people are trying to widely use various solid wastes, with the core goal being recycling. However, up to now, little effort has been invested in establishing relevant standards to guide this utilization. Some minor components in the raw materials (such as magnesium oxide) may have unknown effects on the durability of AAS, which has aroused concern in the industry and deserves further research in the future. There is still controversy over whether some testing methods are applicable or reasonably reflect the true trend of durability (e.g., in tidal environments).

## Figures and Tables

**Table 1:** The impact of acid corrosion on concrete performance.

Types of Acids	Condition	Result	Ref.
Sulfuric acid solution	-	Bubbles are generated on the surface of AAS concrete, accompanied by the smell of rotten eggs; The higher the concentration of sulfuric acid, the more severe the corrosion.	Lanfang [34]
	-	The higher the slag content, the more severe the corrosion.	Lee [36]
	Soak in a 3% concentration solution for 90 days	All specimens showed a significant decrease in compressive strength, but AAS concrete had less strength loss than OPC concrete.	Newaz [45]
	Soak in pH = 1 solution for 120 days	The strength loss of OPC concrete is 71%, and the strength loss of AAS concrete is 21%.	Mithun [46]
	Soak in a 5% concentration solution for 100 days and 365 days	OPC concrete loses 103% of its quality within 100 days, while AASC only loses 10% within 365 days.	Mithun [46]
Phosphoric acid solution, mixed solution of phosphoric acid and sulfuric acid	-	The corrosion effect of phosphoric acid solution is most obvious, while sulfuric acid solution is the least.	Ren [31] Jie [41]
Acetic acid, sulfuric acid solution	Soak at the same pH	The smaller the pH value, the rougher the surface of the sample, and the corrosion effect of acetic acid is the most obvious.	Zhao [43]
Hydrochloric acid solution	-	As the soaking time increases, the quality loss of AAS can reach 4.5–5.5%.	Afridi [50] Hamsashree [51]
	AAS concrete excited by different activators	Low alkali equivalent makes AAS concrete have good acid resistance.	
Sulfuric acid and nitric acid solutions	-	Sulfuric acid has the slowest corrosion effect on AAS cement, while nitric acid has a deeper corrosion depth on AAS than sulfuric acid.	Lloyd [46]
Sulfuric acid, hydrochloric acid, nitric acid solution	Soak at the same concentration of 5%	The compressive strength loss of AAS concrete is highest in sulfuric acid, followed by hydrochloric acid and nitric acid.	Thunuguntla [55]

**Table 2:** The impact of sulfates on the performance of AAS concrete.

Types of Sulfate	Results	Refs.
Na_2_SO_4_ solution	The surface edge of the mortar sample has no obvious damage, and the strength has been lost.	Ahmad [70]
	The soaking and drying cycle was carried out without the formation of gypsum.	Li [71]
	The compressive strength increases while the quality remains basically unchanged.	Guo Jun [72]
	With the continuous extension of erosion time, the compressive strength will still decrease.	Zhu [73,74]
	The surface of the AAS sample remains unchanged.	Ying Hua [78]
	The strength of slag Portland cement first increases slightly and then decreases, while the strength of AAS cement continues to increase during the experimental aging period.	Komljenovic [79]
MgSO_4_ solution	White Mg(OH)_2_ precipitate adheres to the surface of the test specimen.	Ying Hua [78]
	Low concentrations have a relatively small impact on AAS; 5–10% magnesium sulfate can lead to complete disintegration of the gel.	Gong [81]
	The strength loss of AAS concrete is greater than that of OPC concrete.	Komljenovic [80]

**Table 3:** AAS concrete is resistant to seawater corrosion.

Conditions	Results	Refs.
Mixing with seawater	The strength of AAS cement mixed with NaCl increases as the amount of NaCl increases, while the strength of AAS cement mixed with CaCl_2_ does not change significantly.	Jun [89]
	Replacing freshwater with seawater can improve the compressive strength of concrete.	Mengasini [18,90]
Different ambient temperatures.	The higher the ambient temperature, the more pronounced the cracks in the AAS concrete mixed with seawater become.	Li [91]
Using sea sand and seawater for mixing	The hydration product morphology of AAS concrete is influenced; there is a slight increase in drying shrinkage; and the resistance to chloride ion penetration is enhanced.	Yang [100]
	When using water mixed with OPC concrete, initial strength increases, but later strength decreases significantly, and a large amount of corrosion occurs in the steel bars, resulting in corrosion pits.	Dasar [102] Melchers [103] Mohammed [105]
Simulated tidal effect	The samples exposed to simulated tidal zones are more severely damaged than those fully immersed in seawater.	Rashad [101]

## Data Availability

Not applicable.
